# Clostridium myonecrosis — a rare and underdiagnosed condition in the elderly: a case with severe skipping lesions and an overview of treatment guidelines

**DOI:** 10.1186/s12245-022-00458-w

**Published:** 2022-10-05

**Authors:** Ellen Van Asbroeck, Ourania Vasileiadou, Sylvie De Laere, Eddy Van Hedent, Katleen Devue

**Affiliations:** 1 Department of Surgery, ASZ Aalst, Aalst, Belgium; 2 Department of Emergency Medicine, ASZ Aalst, Aalst, Belgium; 3 Department of Vascular Surgery, ASZ Aalst, Aalst, Belgium; 4 Department of Radiology, ASZ Aalst, Aalst, Belgium

**Keywords:** Spontaneous clostridial myonecrosis, Gas gangrene, Clostridial myonecrosis, *Clostridium septicum*

## Abstract

We present an unusual and severe case of spontaneous *clostridial* myonecrosis (SCM) in an elderly patient, with severe skipping lesions spread throughout the body. CT imaging, combined with postmortem available blood cultures, confirmed the diagnosis. We noted an underrepresentation of SCM in the cohort of elderly patients (≥ 85 years), upon a review of case reports in the literature over the last decade. Given the worldwide demographic change resulting in an increase in the number of visits to emergency departments for this age group, it is likely that SCM is underdiagnosed in these elderly patients. This case report aims to increase awareness among emergency physicians to recognize the disease as well as to provide a treatment guideline, in order to provide better care and outcome.

## Introduction

We present an unusual and severe case of spontaneous clostridial myonecrosis, documented by combined radiography and CT scan imaging and confirmed by postmortem blood cultures. Following a literature review in PubMed over the last decade, we found that case reports for clostridial myonecrosis are underrepresented in the cohort of elderly patients (> 75 years). Therefore, we hypothesize that this condition is underdiagnosed in elderly patients.

## Case presentation

Ambulance attendants encountered the patient — an 84-year-old woman living independently at home — lying on the floor of her bedroom and transported her to the emergency department. Upon arrival at 2 pm, the patient was conscious but complained of mild pain all over her body (VAS score of 3/10). Anamnesis did not reveal any particularities in her medical history, allergies, or changes in medication (the patient had been using the anti-arrhythmic drug sotalol for many years).

Vital signs were stable: blood pressure (BP) of 149/61 mm Hg, pulse 60 beats per minute, oxygen saturation of 98%, and body temperature 35.4 °C. Examination of the heart, lungs, and abdomen did not show any particularities. Neurologic examination on arrival showed a normal level of consciousness without any signs of paralysis. The patient presented with normal speech, although communication was difficult because of hearing loss. She appeared alert and oriented but could not remember falling on the floor at her home.

Observations upon physical examination of the patient are as follows:

1. A cold right foot and lower leg with delayed capillary refill and the absence of peripheral pulses
2. Light-reddish coloring on the medial side of the left lower leg, without blisters or palpable crepitus, peripheral pulses were palpable, and the limb had a normal temperature.
3. Painful mobilization of both hips
4. Posterior no signs of infections on the skin, and the spine was not painful at palpation.

Lab results of the arterial blood sample (available within the hour after patient’s arrival) showed abnormal values for hemoglobin (10.5 g/dL), lactate (2.3 mmol/L), urea (80.6 mg/dL), and creatinine (2.2 mg/dL), indicating impaired renal function. After establishing arterial thrombosis of the right leg as the working diagnosis, fluid resuscitation according to a contrast nephropathy scheme was started (glucose 5% with sodium bicarbonate 150 mEQ at 210 ml/h) in order to perform a contrast CT scan of the lower limb. In the mean time, the vascular surgery trainee was informed of the case, and further blood results became available, indicating higher than normal levels of troponin T (0.037 ng/mL), NT-proBNP (34.600 pg/mL), D-dimer (> 8000 ng/mLfib.eq), C-reactive protein (205 mg/L), CK (1757 U/L), LDH (365 U/L), and SGOT (55 U/L). At 5:15 pm, we performed a CT scan with extended range (thorax, abdomen, and limbs) in search of a focus of infection.

**Fig. 1:**
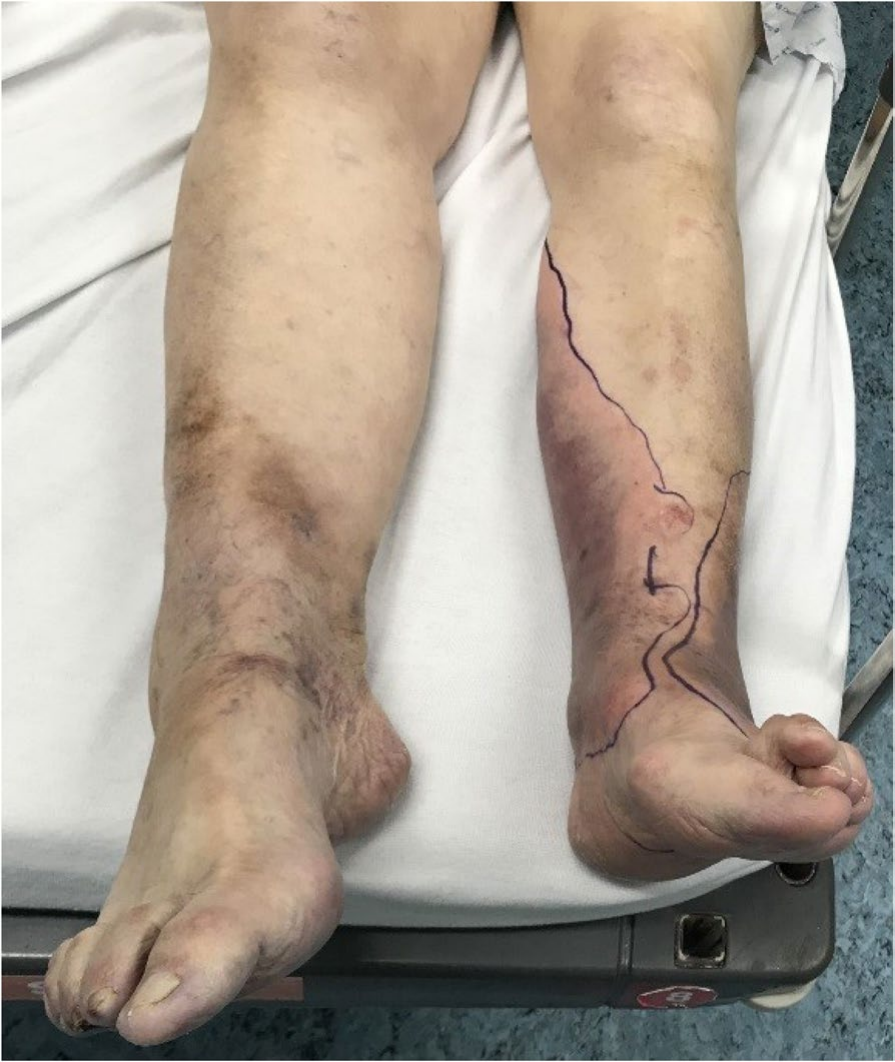
Purple discoloration and bullae formation of the left leg

**Fig. 2:**
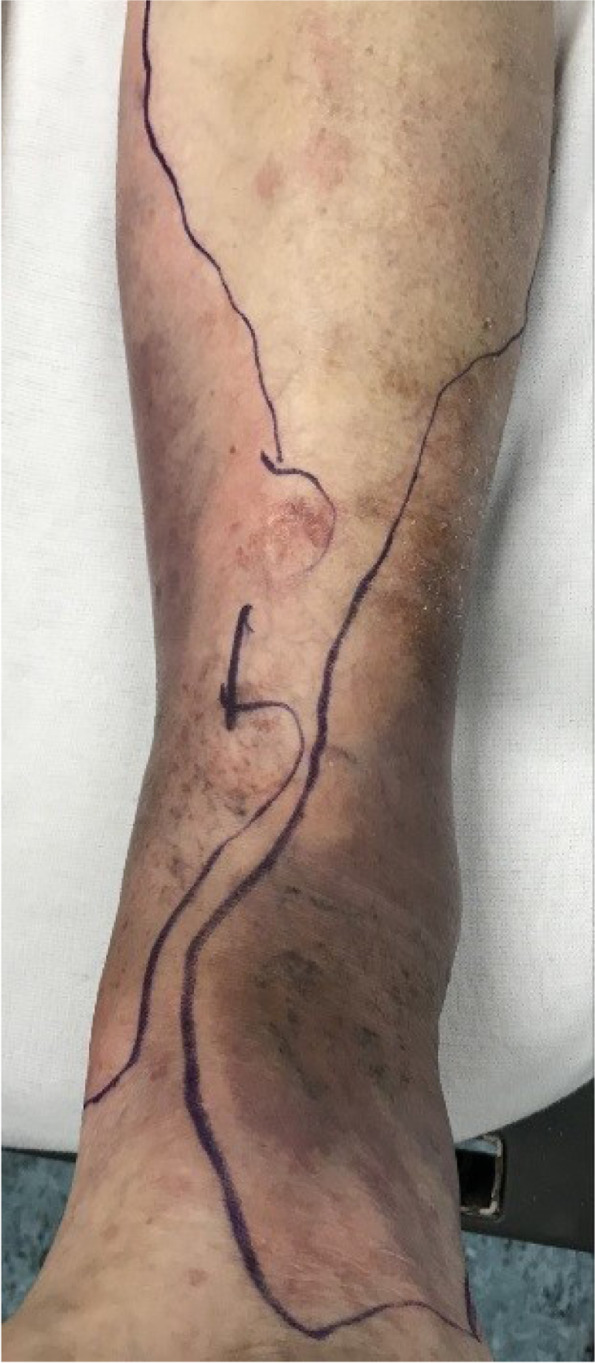
Detailed view of lower left leg

Upon the patient’s return from the radiology department, a vascular surgery trainee performed a clinical re-evaluation. By then, almost 4 h had passed since the patient’s arrival. The skin discoloration on the left leg, initially located on the medial site, was progressing to posterior and to the lateral site, almost causing a circular purple discoloration. Bullae formation was also present, over a well-delineated area (Figs. 1 and 2), still without any signs of crepitation.

**Fig. 3:**
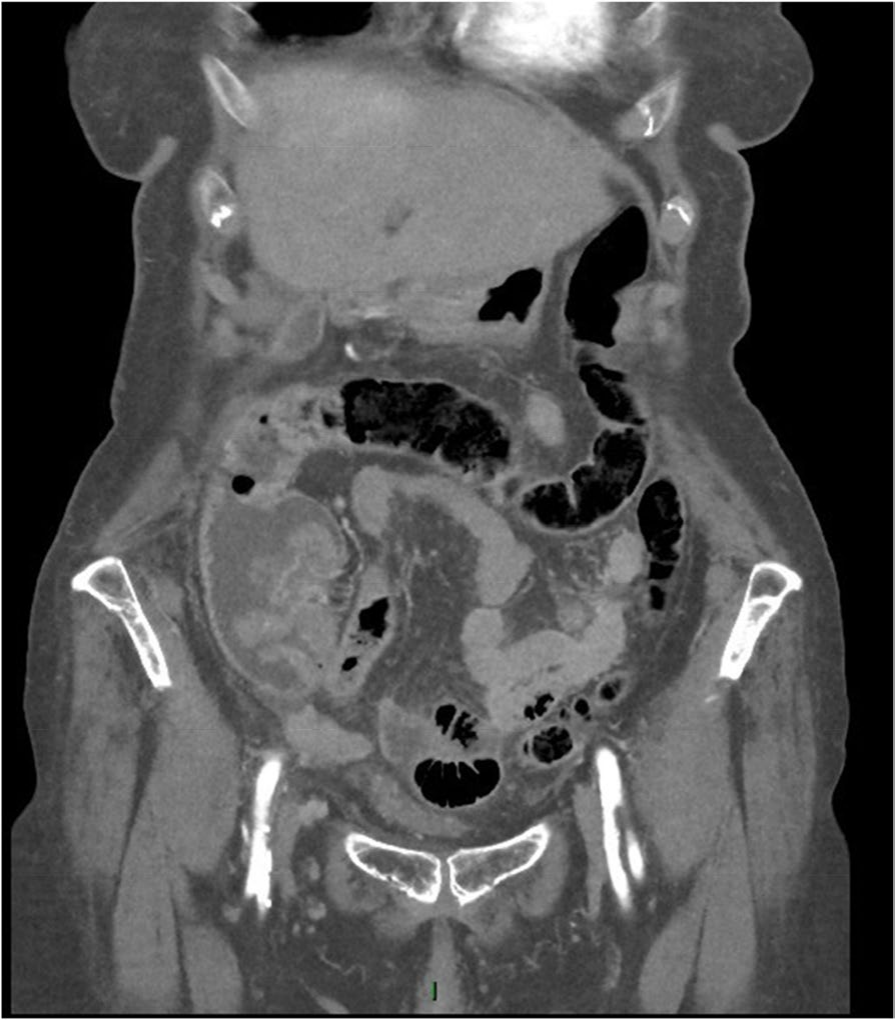
A suspicious big polypus mass in the colon at the valve of Bauhin, possibly ct2N0M0

**Fig. 4:**
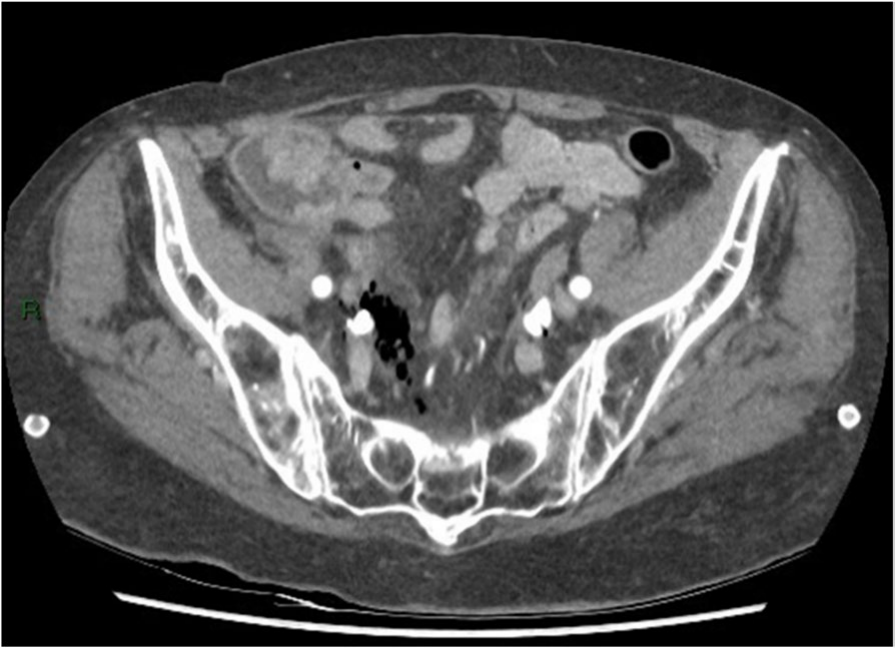
Gas surrounding the iliac vessels on the right side

**Fig. 5:**
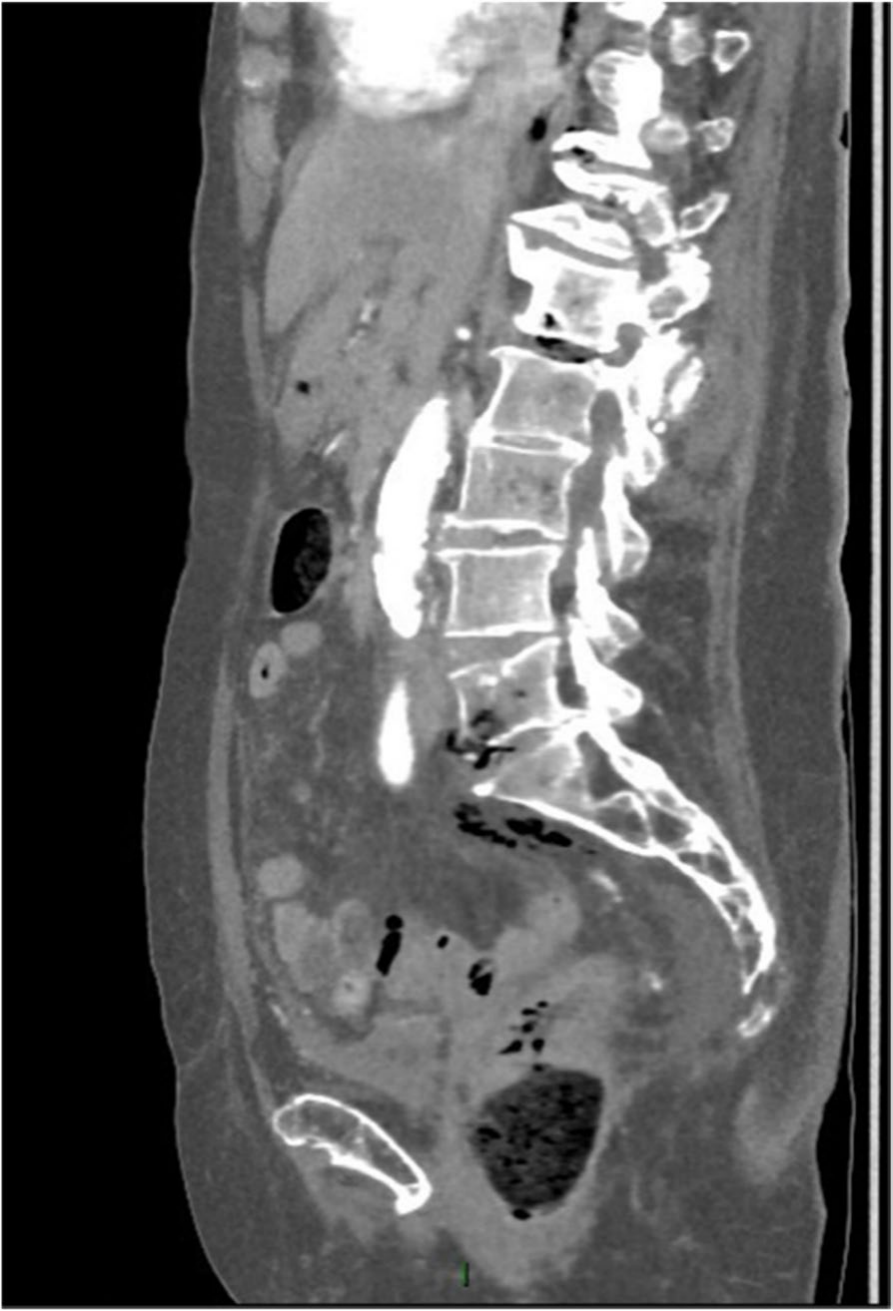
Gas in the retroperitoneal space most profound at the intervertebral space of L5-S1 with spreading along the iliac veins, most remarkable on the right side. Suspicion of localized osteonecrosis in L5-S1

**Fig. 6:**
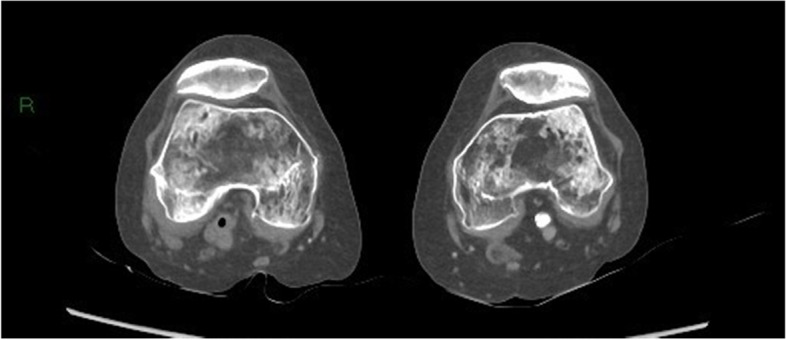
Gas bubble in the popliteal artery on the right side

**Fig. 7:**
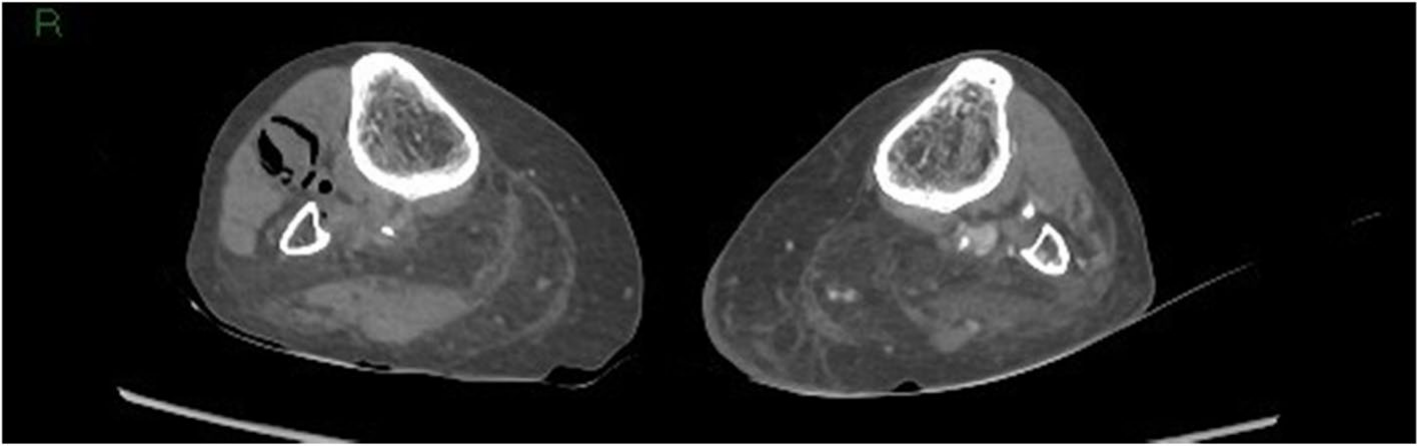
Gas in the right lower leg muscle compartment

**Fig. 8:**
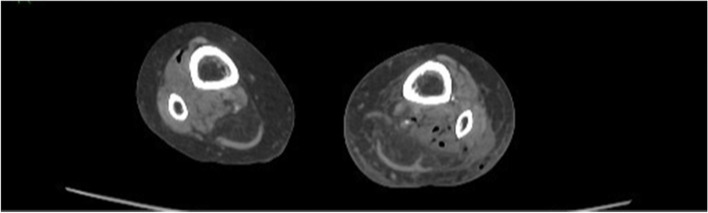
Gas in both leg muscle compartments

Subsequently, the patient was administered 1 g amoxicillin/clavulanic acid, intravenously. Neurological re-evaluation showed lessened responsiveness, disorientation, and confusion (GSC: 14/15, E4, V4, M6). A mere 30 min later, the images of the CT scan became available for interpretation by the vascular surgeon at the emergency department. The CT scan showed several abnormalities, illustrated in Figs. 3, 4, 5, 6, 7 and 8; their respective descriptions are listed below each figure.

Given the rapid evolution of the skin on the left lower calf in combination with gas formation in the muscle compartments (Figs. 6, 7, and 8) and retroperitoneal space, our first working hypothesis of arterial thrombosis was abandoned. The clinical situation likely originated from an anaerobic infection, most probably with a *Clostridium* strain. We found no clear focus of entry, nor visible external wounds. The spreading of gas surrounding the iliac vessels and inside the distal superficial femoral artery on the right side and retroperitoneal space (Figs. 4 and 5) prompted us to broaden our differential diagnosis. We considered a possible necrotizing fasciitis due to a group A *Streptococcus* pathogen, or a retroperitoneal perforation of the de novo found colon tumor (Fig. 3), or a complicated spondylodiscitis (Fig. 5) with hematogenous spreading of emboli. Other — less likely — options were pyomyositis, which causes muscle abscesses mostly by *Staphylococcus. aureus*, or viral myositis and rhabdomyolysis.

While discussing possible differential diagnosis and therapeutic options, the patient’s condition deteriorated further with a GCS of 8 (she only opened her eyes in response to pain stimulus, E2; verbal sounds were incomprehensible, V2; she still moved to localize pain, M5), anisocoria, deviation of the eyes to the right side, and Cheyne-Stokes breathing. Blood pressure stayed stable for a long time but eventually dropped to 90/50 mmHg with a heart rate of 70 beats per minute and oxygen saturation of 94%. Still, there was no fever. Arterial blood sampling showed elevating lactate levels to 3.6 mmol/L, pH level of 7.25, and pO2 level of 58.8 mmHg.

A multidisciplinary team consisting of the emergency physician, the vascular surgeon, and the neurosurgeon on call decided against intervention with surgical debridement because of the multiple distant spread gas seen on the CT scan images. Because of her rapidly declining state and the lack of therapeutic options, additional investigations such as a skin biopsy of the left leg and a CT scan of the brain to visualize air were not executed. In consensus with the patient’s family, we initiated a palliative comfort treatment. The patient died 10 h after arrival in the hospital. Blood cultures, available postmortem, revealed *Clostridium septicum* as the pathogenic culprit and confirmed our diagnosis of spontaneous myonecrosis.

## Review

### Pathogenesis and diagnosis

Historically, myonecrosis (better known as gas gangrene) was a common wound infection due to poor hygiene, with an incidence as high as 5% (currently estimated at 0.1%).

Two distinct types of *clostridial* myonecrosis are known. One type occurs in traumatized tissue and is associated with *Clostridium perfringens* and *Clostridium histolyticum*. The other type arises spontaneously and is caused by *Clostridium septicum*, presenting with hematogenous seeding from the gastrointestinal tract to the muscles. The incidence of spontaneous clostridium myonecrosis (SCM) is not known, probably due to difficult diagnosis. *C. septicum* is estimated to cause 20% of all (traumatic and spontaneous) cases [1]. *Clostridium* species are anaerobic, large, gram-positive, gas-producing rod-like bacteria with the ability to produce environmentally resistant endospores that are widespread in nature and can be found in soil and the intestinal tracts of humans and animals [2]. Still, *C. septicum* is considered a rare component of the gastrointestinal flora, only present in 2% of the population, preferably localized in the cecum and ileocecal junction [3]. Its capacity to cause disease likely originates from coincidental occurrence of transient carriage and enhanced host susceptibility [4].

*C. septicum* produces multiple toxins, of which the alpha toxin, a necrotizing pore-forming cytolysin, is the most lethal, causing the extensive myonecrosis associated with fulminant SCM [5]. The capability to infect normal healthy tissue, due to its aerotolerant characteristics, distinguishes *C. septicum* from other strains such as *C. perfringens* and *C. difficile*, which are obligate anaerobes [6].

The pathogenesis of SCM remains largely unclear, yet three possible patterns have been described: (i) visceral anaerobic cellulitis, (ii) visceral anaerobic cellulitis with contiguous spread to adjacent muscle, and (iii) myonecrosis arising at a site distant from the initial visceral lesion (less common) [7]. The present case entails myonecrosis at multiple distant sites. It is suspected that the infection descends along the iliopsoas sheath from (most often) a gastrointestinal mucosal origin lesion, with ensuing hematogenous spreading [8]. It is well described in literature that such gastrointestinal lesions are frequently undiagnosed adenocarcinoma of the colon, as is the case in our casus [9]. Other predisposing factors such as leukemia, inflammatory bowel disease, diverticulitis, gastrointestinal surgery, lymphoproliferative disorders, chemotherapy, neutropenia, radiation therapy, AIDS, diabetes mellitus, necrotizing enterocolitis or distal ileitis, and the use of NSAID were not applicable to the current case [10].

While it has been established that *C. difficile* infections disproportionately affect older patients (≥ 65 years of age), such a correlation has not yet been described for *C. septicum* infections. Therefore, we performed a retrospective literature search of *C. septicum* case reports in the PubMed database over the last 10 years to gauge the prevalence of SCM in the elderly population. From a total of 160 case reports, we found 89 reports of adult patients with *C. septicum* infection (graph 1). The prevalence of SCM remains at a low steady level throughout the 4 youngest age groups spanning 18–54 years. Noteworthy, we observed a sudden increase in cases within the age group of 55–66 years, persisting up to the group of 74–85 years. In contrast, SCM prevalence in the age group ≥ 85 years dropped significantly, almost to the level found for the youngest age groups. Based on these findings, we hypothesize that SCM is underdiagnosed in the oldest age group.

Early recognition and treatment of SCM are critical for a desired outcome, but diagnosis is not straightforward and is usually delayed [11]. The clinical presentation of SCM often starts with a sudden onset of severe localized muscle pain, in the absence of manifest injury, or other explanatory causes. Sometimes, heaviness or numbness is mentioned as early signs [12]. The skin over the affected area may appear pale at first and then progress rapidly to a bronze appearance and finally to a purple or red hue [13]. Typically, such colorations are combined with the development of edema and bullae filled with cloudy hemorrhagic or purplish fluid. Very often, crepitations are present, which differentiates SCM from streptococcal myositis [1, 14]. Other differential diagnosis can be (i) viral myositis, where injury is also absent but where pain perception is mostly diffuse, rather than localized, or (ii) rhabdomyolysis caused by trauma, intoxication, or metabolic disorders.

Initial systemic manifestations vary from the presence of fever and tachycardia to the development of signs of systemic shock. Illness progresses with thrombocytopenia, anemia, diffuse intravascular coagulation, kidney failure, and acute respiratory distress syndrome, ultimately leading to multiple organ failure [13]. Sometimes, the disease can be obscure and only present with initial tachycardia, whereas severe pain, hypotension, and fever occur later in time [9]. Routine laboratory analyses should be performed, since the levels of creatine phosphokinase, myoglobin, and potassium can be elevated after release from tissue and muscle breakdown. Additionally, knowledge of ABG, lactate, and pre-calcitonin levels can be useful when evaluating sepsis. Imaging such as radiography (echography), CT scan, or MRI may visualize gas bubbles present in soft tissue [1]. Visualized gas offers a differential diagnosis from pyomyositis, where there is usually no systemic toxicity or gas present. On the short term, the involvement of a bacterial pathogen can be proven by gram staining of the affected tissue (e.g., bullous fluid) [14]. If muscle necrosis is present, with spreading to skin, fat, subcutaneous tissue, and fascia, large gram-positive bacilli can be microscopically visible in between the degenerating muscle bundles without the characteristic lack of inflammatory cells (leukocytes) [12]. A final diagnosis can only be obtained from a blood culture that can identify *C. septicum*.

### Treatment and prognosis

There are two options for SCM treatment: (i) early and aggressive, often multiple, surgical debridement and (ii) antibiotic therapy [15]. In want of the identification of the causative pathogen, a broad-spectrum empiric antibiotic treatment should be started promptly. Mostly, piperacillin-tazobactam 4.5 g plus clindamycin 900 mg are administered intravenously every 8 h [16].

Thorough surgical debridement is believed to be the single best predictor of outcome [13], but is not feasible with a widespread infection. In case of elevated compartment pressures, a fasciotomy may be necessary [1].

Once the definitive causative species *C. septicum* is known, the antibiotic therapy can be switched to penicillin 3–4 million units plus clindamycin 900 mg both intravenously every 8 h or tetracycline 500 mg intravenously every 6 h. In case of penicillin allergy, only clindamycin can be used.

Despite the fact that *C. septicum* is aerotolerant, one study did show promising results combining the above treatment with hyperbaric chamber therapy, showing a decrease in mortality from 70 to 25% [9]. However, choosing for hyperbaric oxygen therapy should never delay antibiotics admission or surgical debridement.

Every patient that survives the treatment should undergo a colonoscopy to rule out gastrointestinal tract lesions [16], since a spontaneous systemic infection with *C. septicum* likely originates from infiltration of the pathogen from the gut. If applicable, a colectomy or lesion resection could prevent reinfections from occurring.

Overall prognosis is poor, and mortality rates reach levels of 67–100%. In most cases, death occurs within 24 h [17]. More specifically, risk factors for a fatal outcome are underlying malignancy and an immunocompromised state. The prognosis of SCM is worse compared to the traumatic type, with survival rates as low as 19% [14].

## Discussion and ethical dilemma

Spontaneous myonecrosis after infection with *C. septicum* is a rare diagnosis with insidious clinical manifestation, for which correct treatment often comes too late. Our patient did not present as a textbook example of SCM. Despite the rapid neurological decline and skin discoloration at the lower left leg, she had no fever, nor clear signs of systemic toxicity. Upon arrival at the emergency ward, the patient complained about pain “everywhere in her body.” The pain in both lower legs and back, suffering at flexion of hips, could be explained by the presence of gas bubbles in the bilateral iliopsoas region. Given the patient’s advanced age, she showed a remarkably long hemodynamic stability, prior to the rapid deterioration. The condition of the left lower leg raised no suspicion at the initial admission time but changed fast with formation of bullae and discoloration. The absence of crepitus was in line with the lack of subcutaneous gas seen after CT imaging. Previous case reports mention the presence of gas in the iliac vein, much like the gas bubbles we found in the distal superficial femoral artery on the right side. Gas was also present retroperitoneal, both in between vertebral discs and bone, as in the vessels of both legs. We believe that the spread of gas to multiple skipping or distant intra-arterial and retroperitoneal sites makes our case unique and contributes to an ethical dilemma. The diffuse spreading of bubbles did not allow surgical life-sparing treatment. If the gas would have solely been localized in the leg(s), we could have opted for surgical debridement or amputation. Given the patient’s rapid neurological decline, it was highly likely that the central nervous system was also implicated.

The advanced age of the patient raises the ethical dilemma whether to proceed with an invasive (mutilating) therapy, or not, especially when patients are no longer able to decide if they want such life-changing treatment. The present case did not pose such a dilemma because the patient could not undergo a thorough debridement. Because admission at the ICU with intravenous antibiotics would not have changed the outcome given the advanced disease progression, the only remaining option was palliative care.

## Conclusion

Every day, geriatric patients report to an emergency department after a fall without major consequences. According to our literature survey, spontaneous clostridial myonecrosis is an underestimated condition in this cohort of patients. Since a thorough differential diagnosis and a timely appropriate treatment are crucial for the outcome of SCM, we hope that the current case report heightens awareness among medical staff. The widespread of the presence of gas bubbles throughout the body makes the present case unique and substantiates our decision for palliative care, because no surgical life-sparing treatment was possible. It is our hope that improved recognition of this disease can contribute to a better care and outcome in comparable, less desperate cases.

## Data Availability

All data generated or analyzed during this study are included in this published article.

## Abbreviations

ED: Emergency department
SCM: Spontaneous clostridial myonecrosis
CT scan: Computed tomography scan
ABG: Arterial blood gas
